# Does additional monitoring status increase the reporting of adverse drug reactions? An interrupted time series analysis of EudraVigilance data

**DOI:** 10.1002/pds.5174

**Published:** 2020-12-08

**Authors:** Andrej Segec, Jim Slattery, Daniel R. Morales, Justina Januskiene, Xavier Kurz, Peter Arlett

**Affiliations:** ^1^ Pharmacovigilance and Epidemiology Department European Medicines Agency Amsterdam The Netherlands; ^2^ Division of Population Health and Genomics University of Dundee Dundee UK; ^3^ London School of Hygiene & Tropical Medicine London UK

**Keywords:** additional monitoring, adverse drug reactions, impact, pharmacovigilance

## Abstract

**Purpose:**

To evaluate the impact of including a medicine in the list of medicinal products subject to additional monitoring (AM) on the reporting of adverse drug reactions (ADRs) in the european economic area (EEA).

**Methods:**

Interrupted time series using the monthly number of EEA ADR reports in EudraVigilance during 12 months before and after the addition to AM list. The main outcome was the change (%) in reporting of ADRs with step change as the *a priori* impact model. Further time series analysis was performed using Joinpoint Regression.

**Results:**

The analysis included 11 active substances. No significant immediate (step change) increase of reporting was identified for any product at time of addition to AM list. We identified a significant gradual increase of ADR reporting after addition to AM list (slope change) for two out of five new products—boceprevir (10% per month, 95% confidence interval (CI) 3%–18%) and denosumab‐Xgeva (13% per month, 95% CI 4%–22%). No change was identified for Prolia, another denosumab‐containing product not subject to AM. No significant increase was identified for any product included in the AM list due to the requirement to conduct a PASS. Conversely, a gradual decrease in reporting was identified for natalizumab (−5% per month; 95% CI −10% to −1%), rivaroxaban (−5%; −8 to −3%), and varenicline (−16%; −21 to −10%). The results were corroborated by the Joinpoint analyses, which yielded similar results.

**Conclusions:**

We identified limited evidence that reporting of ADRs increased modestly and gradually for some new products and not for products with PASS requirement.

**KEY POINTS**

- EU legislation mandates that new medicines including new biologicals, medicines authorised subject to conditions or under exceptional circumstances and medicines with the obligation to conduct a post‐authorisation safety study (PASS) are subject to additional monitoring aimed at increasing the reporting of ADRs by patients and healthcare providers.
- The real impact of additional monitoring on reporting of ADRs is currently unknown.
- Using an interrupted time series analysis of EudraVigilance ADR reporting data, we identified limited evidence that reporting of ADRs increased modestly and gradually for some new products. In contrast, reporting of ADRs did not increase (or even decreased) for products subject to AM due to the requirement to conduct a PASS.
- Further work is required to determine the effectiveness of AM as a policy intervention.

## 1 INTRODUCTION

Pharmacovigilance is the science and activities relating to the detection, assessment, understanding and prevention of adverse drug reactions (ADRs) or any other medicine‐related problem. Pharmacovigilance contributes to the protection and promotion of public health, timely access to innovative medicines and their safe and effective use after authorisation. Reporting of suspected ADRs is the cornerstone of pharmacovigilance as regular monitoring of newly received reports allows the identification of new risks and changes in known risks for medicines. Additional monitoring (AM) was introduced in Europe in 2010, following the revision of pharmacovigilance legislation (Regulation [EC] No 726/2004, Article 23), to increase the reporting of ADRs for targeted medicines.^1^, ^2^

Medicines falling under the mandatory scope of additional monitoring include new medicines, new biologicals, medicines authorised subject to conditions or under exceptional circumstances, and medicines with the obligation to conduct a post‐authorisation safety study (PASS). Such medicines are identified with an inverted black triangle and an accompanying explanatory statement in their product information and are included in the list of medicines under additional monitoring which is updated and published monthly (AM list). Once on the list, a medicine will remain under additional monitoring for 5 years or until removed from the list by the Pharmacovigilance Risk Assessment Committee (PRAC). The black triangle allows to quickly identify medicines that are subject to additional monitoring and encourages patients and healthcare professionals to report any ADRs. At the end of 2019, 342 medicines were included in the AM list.^2^, ^3^, ^4^

The impact of additional monitoring as a policy intervention on the reporting of ADRs is unknown. Measuring the impact of regulatory decisions is important for all pharmacovigilance activities in order to improve existing processes.^5^, ^6^ Consequently, we undertook this study using EudraVigilance data to investigate whether the inclusion of medicines in the AM list increases the reporting of ADRs for those medicines in Europe. The study was part of a data gathering project on the experience with additional monitoring, together with a survey of patients' and healthcare professionals' attitudes and behaviours towards reporting ADRs.^1^, ^7^

## 2 METHODS

### 2.1 Data sources

Information on the number of reported ADRs was obtained from EudraVigilance, the European database of ADR reports maintained by the european medicines agency (EMA) on behalf of the EU network, which at the end of 2018 contained over 14.5 million reports of suspected ADRs.^8^, ^9^, ^10^

### 2.2 Exposures

Eligible medicines were identified from the December 2015 version of the AM list,^3^ by excluding those which did not have 12 months of authorised use prior to their addition to the list (to ensure we had a baseline of reporting for comparison) and those medicines for which their monthly reporting of ADRs from the EEA was less than 10 reports (over the 24 months of observation).

For each substance, we obtained the monthly number of post‐marketing individual case reports of ADRs originating from the EEA from EudraVigilance,^8^, ^9^, ^10^ the EU database of ADR reports, for 12 months prior to and 12 months after inclusion of the product in the AM list (the intervention).

To account for changes in the number of patients exposed to the medicines over time, we calculated the size of at‐risk population using medicine consumption/exposure data estimates obtained from Periodic Safety Update Reports^11^ (PSUR) held by EMA for centrally‐authorised products. Exposure data are normally reported as the total person‐years exposed during the interval covered by the PSUR which can range from 6 months to several years. We therefore divided the person‐years by the number of years covered by a PSUR period and smoothed the data over time using a piecewise linear form between the mid‐point intervals. Where PSUR data were not available for the whole period under observation, but was missing only for a few months, we imputed the modelled exposure to the missing months based on the period with available data, as a continuation of that trendline. For products for which PSUR data was not available, we substituted the estimates of exposure by defined daily dose from publicly available nationwide drug consumption databases available from Denmark,^12^ France,^13^ Netherlands,^14^ Norway,^15^ Sweden^16^ and United Kingdom,^17^ representing approx. 30% of the EU population.

### 2.3 Analysis

Interrupted time series (ITS) is a suitable analytical method for evaluating the effect of interventions implemented at population level at a defined time point.^18^ We applied segmented regression analysis to the interrupted time series data using Poisson regression and modelling the count of events per month, whilst offsetting the changes in at‐risk population. To account for over‐dispersion of the data we corrected the standard error by applying a scale parameter based on Pearson chi‐square statistic divided by the residual degrees of freedom and we adjusted for seasonality using Fourier terms with two pairs of sine/cosine functions.^18^, ^19^ We postulated an immediate effect (step change) as our *a priori* impact model; we also investigated a possible gradual change in trend (slope change). STATA (College Station, Texas, USA) version 15 was used for the analyses.

Further time series analysis was performed in parallel using Joinpoint Regression Program^20^ (National Cancer Institute, Maryland, USA) version 4.5.0.1, using the Grid search method and Permutation testing. Statistical analysis using joinpoint regression identifies the time point(s) where a marked change in trend (the ‘joinpoint’) has occurred and estimates the regression function compared with the previously identified joinpoints. As the final number of joinpoints is established on the basis of a statistical criterion and their position is not fixed it does not require that an intervention date is pre‐specified unlike interrupted time series regression.^21^ We compared the results of both statistical analyses.

## 3 RESULTS

We identified 82 eligible products corresponding to 79 substances from the AM list with at least 12 months of baseline data and excluded 68 substances with low ADR reporting. The final analysis therefore contained 11 substances, five of which were included in the AM list due to new substance status (boceprevir, telaprevir, vemurafenib, fingolimod and denosumab) and six that were included because of an imposed PASS (imatinib, lenalidomide, natalizumab, rivaroxaban, valproic acid and varenicline), as detailed in Flowchart 1 and Table 1. Analyses were performed at substance level except for denosumab, for which we identified two products with different indications (Xgeva and Prolia) and discrepant AM status. Xgeva is indicated for the prevention of skeletal related events in adults with advanced malignancies involving the bone and for the treatment of giant cell tumour of bone and is subject to AM.^22^ Prolia is indicated for the treatment of osteoporosis and for bone loss associated with hormone ablation in men with prostate cancer and is not subject to AM.^23^ The analysis of these products was performed separately at product level with Prolia used as a control for Xgeva.

**FLOWCHART 1:**
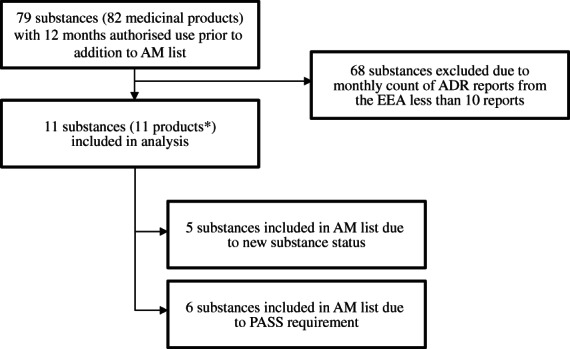
Selection of substances for the study. *Count includes valproic acid as one product and does not include denosumab‐Prolia (used as control)

**TABLE 1:** Characteristics of medicinal products included in the study

Active substance	Product name	Indications/therapeutic area (MeSH)	Date of inclusion in the AM list	Reason for inclusion in the AM list
Boceprevir	Victrelis	Hepatitis C, chronic	April 2013	New active substance
Telaprevir	Incivo	Hepatitis C, chronic	April 2013	New active substance
Vemurafenib	Zelboraf	Melanoma	April 2013	New active substance
Fingolimod	Gilenya	Multiple sclerosis	April 2013	New active substance, PASS
Denosumab	Xgeva	•Fractures, bone •Neoplasm metastasis	April 2013	New biological
	Prolia (control for Xgeva)	•Bone resorption •Osteoporosis, Postmenopausal	N/A	N/A
Imatinib	Glivec	•Precursor cell lymphoblastic leukaemia‐lymphoma •Gastrointestinal stromal tumours •Dermatofibrosarcoma •Myelodysplastic‐myeloproliferative diseases •Leukaemia, myelogenous, chronic, BCR‐ABL positive •Hypereosinophilic syndrome	September 2014	PASS
Lenalidomide	Revlimid	•Multiple myeloma •Lymphoma, mantle‐cell •Myelodysplastic Syndromes	June 2014	PASS
Natalizumab	Tysabri	Multiple sclerosis	April 2013	PASS
Rivaroxaban	Xarelto	•Arthroplasty, replacement •Venous thromboembolism	July 2013	PASS
Valproic acid	Various	•Bipolar disorder •Epilepsy •Migraine disorders	January 2015	PASS
Varenicline	Champix	Tobacco use cessation	April 2013	PASS

**FIGURE 1:**
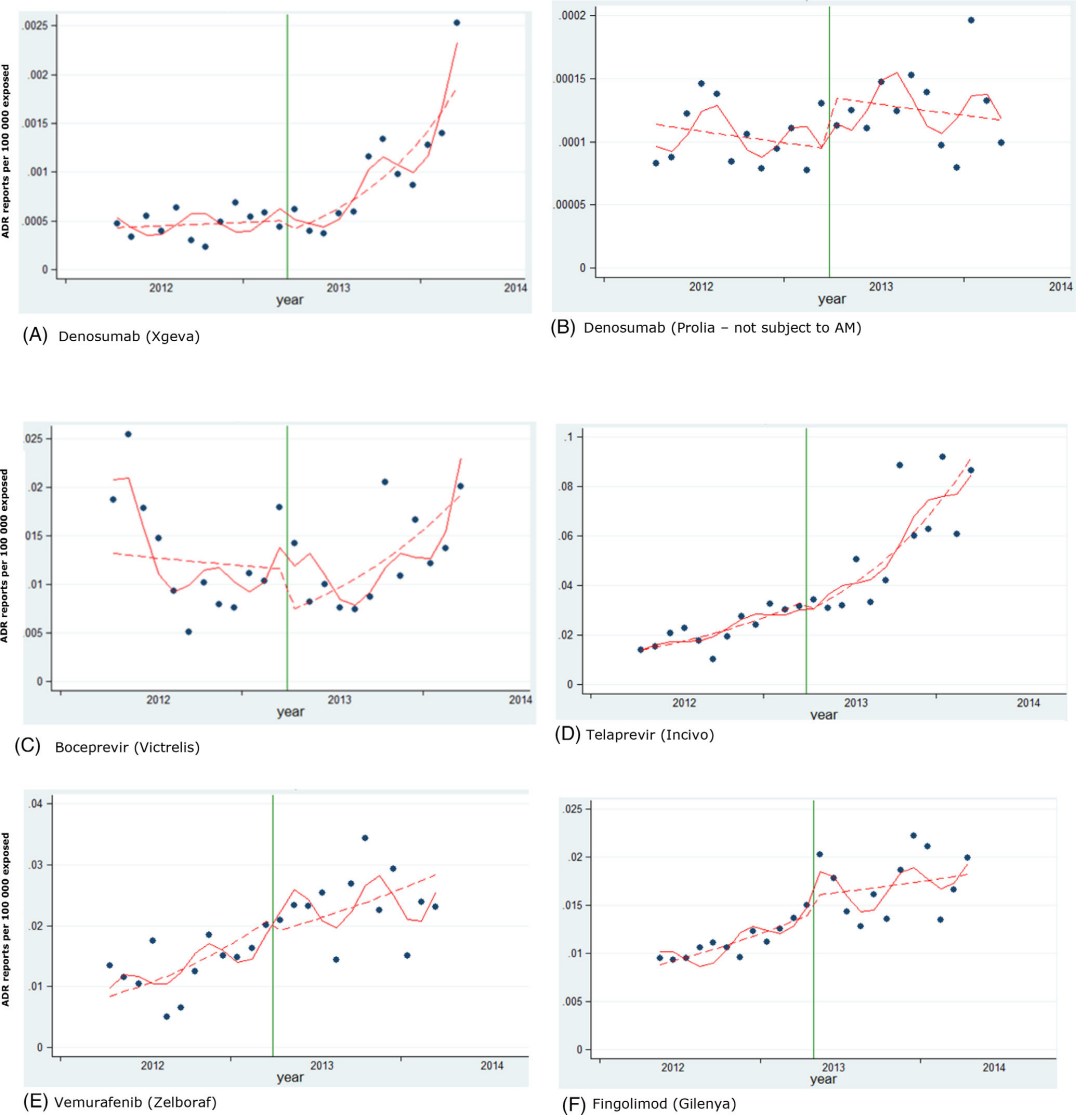
Interrupted time series analysis. New products included in the study. Reporting of post‐marketing ADRs from the EEA. Green vertical line: addition to AM list. Red line: predicted reporting from seasonality‐adjusted model. Dashed line: de‐seasonalised trend [Colour figure can be viewed at wileyonlinelibrary.com]

**FIGURE 2:**
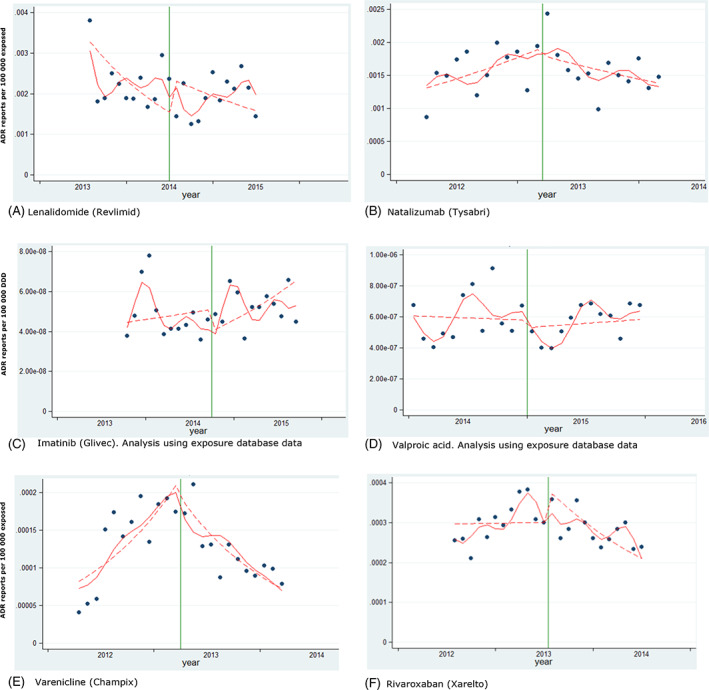
Interrupted time series analysis. Products included in the study due to the requirement to conduct a PASS. Reporting of post‐marketing ADRs from the EEA. Green vertical line: addition to AM list. Red line: predicted reporting from seasonality‐adjusted model. Dashed line: de‐seasonalised trend [Colour figure can be viewed at wileyonlinelibrary.com]

As presented in Table 2 and Figures 1 and 2, we did not identify a significant immediate effect (step change) for any studied product based on ITS analysis. We identified a significant increase of ADR reporting as a gradual change in trend after the addition to AM list (slope change) for boceprevir (10% per month, 95% CI 3%–18%) and denosumab‐Xgeva (13% per month, 95% CI 4%–22%). No significant change in slope was identified for the other new substances (telaprevir, vemurafenib and fingolimod). No significant change (step or slope) was identified for imatinib, lenalidomide and valproate. Conversely, for three products included in the AM list due to the requirement to conduct a PASS, we identified a decrease in the reporting of ADRs after addition to the list (slope decrease): natalizumab (−5% per month, 95% CI −10% to −1%), rivaroxaban (−5% per month, 95% CI −8% to −3%) and varenicline (−16% per month, 95% CI −21− to −10%).

**TABLE 2:** Summary of results—interrupted time series analysis of the monthly reporting of post‐marketing ADRs from the EEA

Active substance	Step change	Slope change
	RR (95% CI)	RR (95% CI)
Boceprevir^a^	0.59 (0.23–1.55)	**1.10 (1.03–1.18)**
Telaprevir^a^	0.85 (0.35–2.06)	1.02 (0.96–1.09)
Vemurafenib^a^	0.90 (0.38–2.11)	0.96 (0.89–1.02)
Fingolimod^a^	1.14 (0.74–1.75)	0.97 (0.94–1.00)
Denosumab^a^—Xgeva	0.73 (0.30–1.77)	**1.13 (1.04–1.22)**
Denosumab^a^—Prolia (not in the AM list, control for Xgeva)	1.43 (0.62–3.28)	1.00 (0.94–1.07)
Imatinib^b^	0.78 (0.42–1.42)	1.03 (0.99–1.08)
Lenalidomide	1.54 (0.71–3.53)	1.03 (0.98–1.10)
Natalizumab	0.96 (0.49–1.87)	**0.95 (0.90–0.99)**
Rivaroxaban	1.30 (0.92–1.85)	**0.95 (0.92–0.97)**
Valproic acid^b^	0.91 (0.50–1.67)	1.01 (0.97–1.06)
Varenicline	0.96 (0.42–2.23)	**0.84 (0.79–0.90)**

^a^ New medicines.

b Analysis using exposure data from prescription databases (PSUR data not available).

When the two denosumab‐containing products were compared, the reporting for Xgeva, a product that was subject to AM, increased by 13% (95% CI 4%–22%) per month, but we detected no significant changes in reporting of ADRs for Prolia (0%, 95% CI −6% to 7%), a product that was not subject to AM.

**FIGURE 3:**
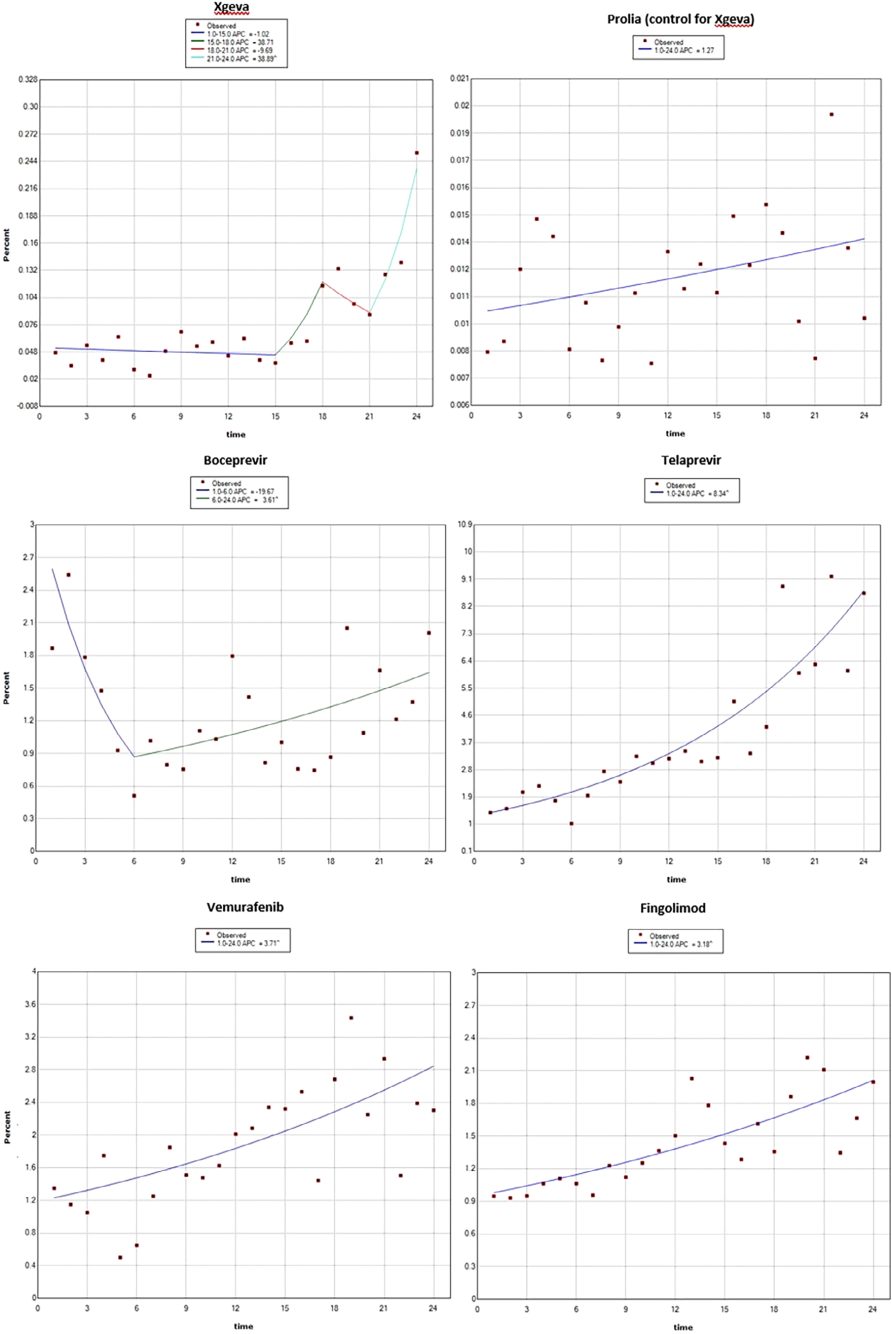
Joinpoint regression analysis—new products included in the study. Reporting of post‐marketing ADRs from the EEA, addition to AM list is at time point 12 (time in months) [Colour figure can be viewed at wileyonlinelibrary.com]

**FIGURE 4:**
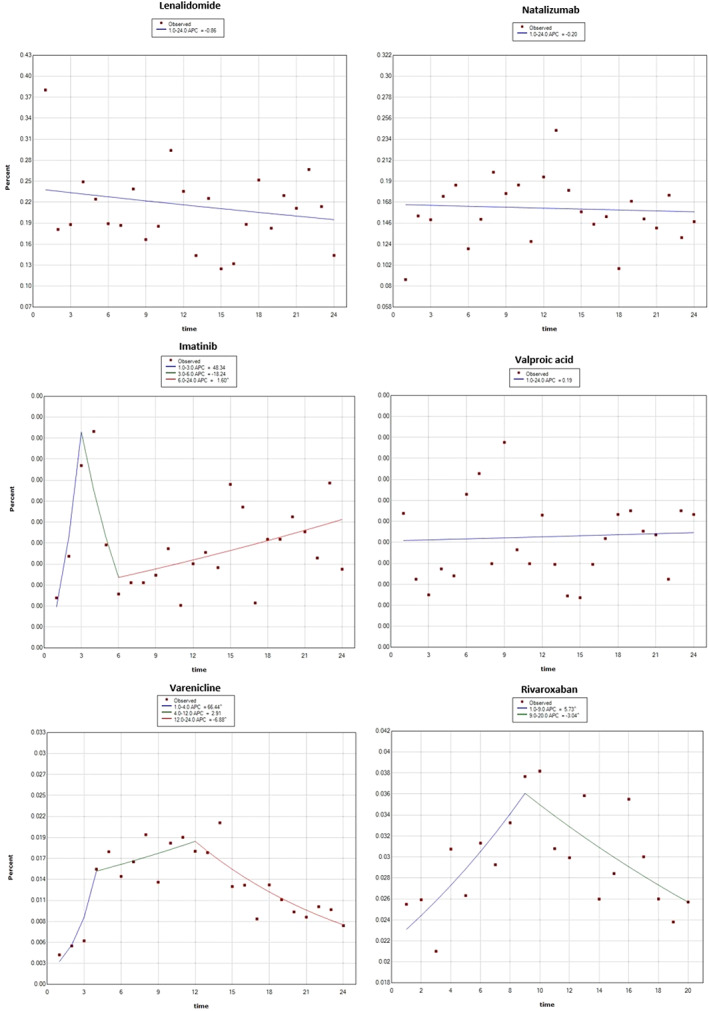
Joinpoint regression analysis—products included in the study due to PASS requirement. Reporting of post‐marketing ADRs from the EEA, addition to AM list is at time point 12 (time in months) [Colour figure can be viewed at wileyonlinelibrary.com]

The results were broadly comparably with the results of the Joinpoint analyses, which are presented in Figures 3 and 4, with the exception of natalizumab, for which no change was identified in the Joinpoint analysis.

## 4 DISCUSSION

In our study we identified that ADR reporting increased after addition to the AM list for two out of five new products, in the order of 10%–13% per month. For products included in the AM list due to the requirement for a PASS we identified either no changes or a decrease in the reporting of ADRs, in the order of −5 and −16% per time point. For denosumab, the only medicine that had two products with differing AM status, we observed a 13% increase in reporting for Xgeva, with no changes observed for Prolia (used as a control for Xgeva), which is compatible with an effect of additional monitoring. The results were broadly comparable with the results of the Joinpoint regression analysis.

This is to our knowledge the first study evaluating the impact of additional monitoring on the reporting of ADRs. Studies have previously reported that ADRs for medicines are under‐reported, including for medicines subject to additional monitoring, and including serious and fatal ADRs.^24^, ^25^, ^26^, ^27^ Such information is vital for pharmacovigilance decision making. Unequal and sometimes low awareness of additional monitoring amongst healthcare professionals was reported as potential reasons for this.^28^ Our separate study of the knowledge of and attitudes to ADR reporting found that although 88% of respondents would report an ADR for a medicine subject to AM, identified with a black triangle, 1 in 5 reporters misunderstood the concept of AM and only 37% of those who reported an ADR for a product subject to AM were influenced by the AM status.^1^ These factors could partially explain the limited effect we found on increasing the reporting of ADRs.

Due to methodological limitations, the maximum number of substances that we were able to include in our study (11 of 79 substances) was limited by the availability of their baseline data or low reporting, which restricts the overall generalisability of the findings. A large number of new medicines were excluded simply because there was no comparative data available before their inclusion in the AM list. The small sample of substances that we were able to study is also limited in terms of chemical/biological and pharmacological classes and may therefore not be universally generalisable to all medicines subject to AM. A possible solution to overcome this limitation would be to stagger future implementation of such policy interventions in various regions over time, which would serve as comparators in a step‐wedge type of approach, but this may not be feasible for EU wide interventions. Alternatively, where possible, a controlled ITS design can be used, as illustrated with the denosumab example. Another approach that could be employed would be to study the change in reporting at the time of removal of products from the AM list.

Our study focused on changes during 12 months after the addition to AM list. For products already on the market which newly acquire additional monitoring status, it may take several months or years for the medicines with the updated product information to reach patients (medicines already distributed are not re‐labelled). It is therefore possible that a longer follow‐up period may be needed to detect significant changes, if awareness of the AM status increases slowly and gradually. Considering that no step change was observed in our study, this remains a possibility. Longer follow up would also allow studying whether any observed increases are sustained or temporary and thus whether immediate changes and long‐term patterns differ. However, as ITS examines associations only, this would be at the risk that other interventions including regulatory interventions, may confound the interpretation over time.

Additionally, the monthly counts of ADR reports were generally low. This combined with 24 time points and unequal variability in the monthly reports, restricted the power to detect a difference, as well as the utility of seasonality adjustment. Due to the small number of substances which we were able to study as well as the low monthly ADR counts we were unable to stratify the analyses by the reason of addition to AM list, by country, or by reporter type (patients or healthcare professionals) as initially intended. Therefore although differences in ADR reporting may exist between different reporter types, as reported in our separate study on reporter attitudes and behaviours,^1^ we could not examine this effect in this study. Seven of the studied substances were included in the first version of the list in April 2013 and the remaining four between 2013 and 2015. Therefore, changes in these reporting sub‐categories and changes over time cannot be excluded.

The application of ITS methods to medicine use data, other than being limited by the lack of baseline data for new medicines, also requires reliable estimates of exposure data due to the often rapid changes in the size of the population at risk, as opposed to population studies. Medicine use can increase rapidly after authorisation/reimbursement and inclusion in clinical guidelines or decrease due to safety concerns or replacement with a more effective, safer or more convenient competitor. The estimates of exposure that we have relied on are often based on approximations from sales data using an expected dose and treatment duration^11^ and can therefore be imprecise and be a source of residual confounding as well as distort important trends.

Other influences in the changes in exposure, especially if they also relate to the reporting of ADRs would be equally obscured by such calculations and cannot thus be investigated. Similarly, the absence of knowledge of other time‐varying confounders and their exact timing, for example, media attention to the results of a study suggesting the risk of serious ADRs, direct healthcare professional communication, restrictions in authorised use due to ADRs, increased awareness due to changes in the international or national treatment guidelines and precautions/screening at treatment initiation or recommendations for regular testing, and so on, which may have influenced the reporting of ADRs, can further complicate the interpretation of time series analyses or lead to unmeasured confounding. Additionally, patients who are at the highest risk of an adverse outcome may no longer be treated after such regulatory actions, and thus the occurrence of ADRs decreases. We did not collect details about the lifecycle of individual medicines included in our study, and therefore couldn't investigate these factors in the current study.

Indeed, half of the substances included in our study were included in the AM list due to the obligation to conduct a PASS. The imposition of a PASS often follows the emergence of serious ADRs, concerns about medicine safety or the evaluation of benefit–risk balance by the PRAC (e.g. via a referral procedure), with consequent media attention. Such a setting is prone to confounding due to other regulatory actions and media attention possibly influencing ADR reporting and therefore the results are difficult to interpret in terms of causality. This may be one reason why we observed a fall in ADR reporting for several products added to the AM list due to an imposed PASS. However, should this observation be confirmed in future research, it may serve to inform any future discussions on the legislation governing additional monitoring.

## 5 CONCLUSION

In summary, we identified limited evidence that reporting of ADRs increased modestly and gradually for some new products and did not increase for products subject to AM due to the requirement to conduct a PASS. The small number of medicines that we were able to include in our study, together with its ecological design, makes the causality of this observation difficult to establish. We suggest that, in the future it would be worthwhile to pre‐specify the methods for the evaluation of policy interventions such as the introduction of additional monitoring, to help overcome the shortcomings of a retrospective evaluation. Given the limitations in our results, we would welcome suggestions from the research and regulatory communities on complementary methods that might be applied to study the impact of AM.

## ETHICAL STATEMENT

The study did not involve human subjects and relied on routine data.

## CONFLICT OF INTEREST

The authors have no conflict of interest to declare.
